# Prevalence and determinants of potentially inappropriate medications in elderly inpatients in Thailand: a retrospective observational study based on the 2019 Beers criteria

**DOI:** 10.1080/20523211.2023.2285958

**Published:** 2023-12-14

**Authors:** Tatta Sriboonruang, Sirichai Chusiri, Jiraphan Ritsamdang

**Affiliations:** Department of Pharmacy Practice, Faculty of Pharmaceutical Sciences, Chulalongkorn University, Bangkok, Thailand

**Keywords:** Potentially inappropriate medications, Beers criteria, length of stay, inpatient, elderly, Thailand

## Abstract

**Background:**

The prevalence of potentially inappropriate medications (PIMs), including NSAIDs, first-generation antihistamines, tricyclic antidepressants (TCAs), and benzodiazepines among elderly inpatients in Thailand, based on the 2019 Beers criteria, is insufficiently investigated.

**Methods:**

This study retrospectively examined 300 elderly patients in a Thai tertiary hospital, assessing four PIM classes based on the 2019 Beers criteria and exploring factors and variations in PIM prescription patterns across different phases of hospitalisation.

**Results:**

The study found an overall PIM prescription rate of 28%, consisting of: benzodiazepines (14%), first-generation antihistamines (9%), NSAIDs (3%), and TCAs (2%). Patients taking at least 5 medications prior to admission were more likely to receive PIMs (OR 3.77, 95% CI 1.15–12.35). Furthermore, PIM prescription was significantly associated with age, showing a 4.8% yearly increase (*p* = 0.01), and the number of comorbidities increased by 16.2% per unit (*p* = 0.021). Additionally, PIM use during admission was significantly linked to a longer hospital stay (OR 3.32, 95% CI 1.50–7.33).

**Conclusions:**

These findings emphasise the need for continued monitoring and optimisation of medication management, and collaboration between pharmacists and physicians to review and adjust prescriptions, especially in elderly inpatients experiencing polypharmacy and multiple comorbidities.

## Background

Potentially inappropriate medications (PIMs), particularly benzodiazepines (BZDs), first-generation antihistamines, non-steroidal anti-inflammatory drugs (NSAIDs), and tricyclic antidepressants (TCAs), present significant risks to geriatric populations due to altered pharmacokinetics and pharmacodynamics. (By the American Geriatrics Society 2015 Beers Criteria Update Expert Panel, 2015) These medications have been implicated in adverse outcomes, including but not limited to cognitive impairment, gastrointestinal hemorrhage, falls, and fractures (Budnitz et al., 2011).

The global prevalence of PIMs in geriatrics exhibits considerable variability, ranging from 24% to 40%. (Hamilton et al., 2009) This phenomenon transcends geographical boundaries, as corroborated by studies conducted in nations including Nepal and Italy (Napolitano et al., 2013; Poudel et al., 2019).

The administration of these medications to hospitalised geriatric patients exacerbates existing risks. For example, NSAIDs are associated with gastrointestinal and renal complications, while first-generation antihistamines, TCAs, and BZDs contribute to cognitive decline and increased fall risk. (O'Sullivan et al., 2014; Rudolph et al., 2008; van der Cammen et al., 2014) The 2019 update of the Beers criteria serves as a pivotal resource for healthcare providers in mitigating these risks (Alldred et al., 2016; By the 2019 American Geriatrics Society Beers Criteria® Update Expert Panel, 2019).

In the context of Thailand, extant literature reports a PIM prevalence as high as 63.98% across healthcare settings. (Varavithya et al., 2022) However, a discernible research gap exists in the evaluation of these four classes of PIMs in Thai inpatient settings, particularly in alignment with the 2019 Beers criteria. This study aims to explore the prevalence and determinants of these PIMs within internal medicine wards of a tertiary university hospital in Bangkok, Thailand.

## Methods

### Study design and setting

This retrospective observational study utilised the 2019 Beers criteria to estimate the prevalence of four commonly prescribed PIMs – benzodiazepines, first-generation antihistamines, NSAIDs, and TCAs – in elderly patients admitted to internal medicine wards at a tertiary university hospital in Bangkok, Thailand, between July 2018 and September 2019.

### Study population

The study population consisted of patients aged 65 years or older admitted to internal medicine wards during the study period. Inclusion criteria were patients aged at least 65 years, while exclusion criteria were: 1) patients with a history of referral to the intensive care unit during admission, 2) patients receiving palliative care, and 3) patients with hometowns or nationalities in countries outside of Asia.

### Sample size calculation

The sample size for this study was calculated with two distinct objectives, each requiring a different sample size. A minimum of 100 patients was deemed sufficient for assessing the prevalence of PIMs. For the logistic regression analyses aimed at identifying 14 potential predictive factors, a sample size of 280 was ideal, adhering to the ‘rule of 20n,’ which recommends a minimum of 20 observations per predictor variable. To account for potential data loss during the study, a sample size of 300 was established, for which ethical approval was obtained.

### Data collection

Data for this study were retrospectively extracted from electronic medical records and systematically entered a case report form designed by the research team. The case report form was divided into two main sections: 1) patient demographics and clinical characteristics and 2) data on medication use and relevant laboratory values.

### Data collection and variable assessment

The research gathered an array of variables to discern the determinants influencing the prescription of PIMs in an elderly population. These variables encompassed demographic and clinical factors such as age, body mass index (BMI), healthcare coverage, the number of diagnosed comorbidities, duration of hospitalisation, history of drug allergies, and the utilisation of medications both prior to and during the hospital stay.

### Statistical methodology

Descriptive statistical methods were employed to delineate the characteristics of the study sample. Additionally, the prevalence rates for each of the four scrutinised medication categories—NSAIDs, first-generation antihistamines, TCAs, and BZDs—were computed. Comparative analyses between patients receiving PIMs and those not receiving PIMs were conducted using Independent t-tests and Mann–Whitney tests.

- Prevalence Estimation: The prevalence of PIM usage among the elderly was ascertained for each of the four medication classes under investigation. The prevalence rate was computed using the equation: Prevalence = (No. of patients with PIM prescription) / (Total number of patients) x 100
- Determinant Identification: To identify potential determinants of PIM use, we conducted bivariate analyses on 14 predefined variables: age, BMI, marital status, occupation, healthcare coverage, number of comorbidities, duration of hospital stay, history of drug allergies, tobacco usage, alcohol usage, medication count before hospitalisation, medication count during hospitalisation, medication count at discharge, and history of herbs and supplement use. Variables demonstrating a statistically significant association with PIM use, identified by a p-value of less than 0.20, or those deemed clinically relevant, were advanced for further analysis. Subsequently, we employed a multiple binary logistic regression model to further investigate the relationship between these variables and PIM use. Utilising a stepwise selection process, variables were successively eliminated from the model based on the criterion of the highest p-value. The model's goodness-of-fit at each step was assessed using the likelihood-ratio test.

Statistical tests were considered significant at a p-value of 0.05. IBM SPSS Statistics for Windows, version 22.0 (IBM Corp., Armonk, N.Y., USA), Chulalongkorn University licensed, was used for the analysis.

## Results

### Sample characteristics

In this study, we analysed the characteristics of 300 patients (150 males, 150 females) with a mean age of 78.08 ± 8.3 years (Table 1). Most participants (63%) were classified as late old (≥75 years) and the rest (37%) as early old (65–74 years). All patients had at least one comorbidity, with 80.33% having four or more comorbidities. The median number of comorbidities was 5 (range 4-7). Cardiovascular disease was the most common comorbidity (233 patients), followed by endocrine disease (140 patients) and infectious disease (107 patients). Further details on the participants’ characteristics, such as body mass index, marital status, occupation, and health care scheme, are provided in the supplementary material [See Supplementary Table S1, Additional File 1].

**Table 1.** Patient characteristics (*n* = 300).

Characteristics	Number (%)
Mean age ± SD (years)	78.08 ± 8.3
Age range	
Early old age (65-74 years)	111 (37)
Late old age (at least 75 years)	189 (63)
Number of comorbidities	
None	0 (0.00)
1 disease	7 (2.33)
2 diseases	16 (5.33)
3 diseases	36 (12.00)
≥ 4 diseases	241 (80.33)

### PIM prescription patterns

Overall, the prevalence of PIMs was 28% in the total cohort (n = 85/300), according to the 2019 Beers criteria. The data indicated that BZDs were the most frequently prescribed PIM class, accounting for 14% of the cohort, with 42 patients receiving them (Table 2). Among BZDs, lorazepam was the most prevalent BZD, prescribed to 35 patients. Notably, two patients received multiple BZDs concurrently prior to admission. First-generation antihistamines ranked second, constituting 9% of the cohort; chlorpheniramine was the most frequently prescribed. One patient received two different first-generation antihistamines, and another received three items concurrently, all of which were newly initiated during their hospital stay, with no overlap with pre-existing prescriptions. NSAIDs accounted for 3% of the cohort, followed by TCAs at 2%.

**Table 2.** Prevalence of PIMs among study participants (*n* = 300).

Medication Category	No. of Patients (%)	Specific Drugs
Benzodiazepines	42 (14%)	Lorazepam (35), Clonazepam (5), Alprazolam (3), Estazolam (1)
First-generation antihistamines	28 (9%)	Chlorpheniramine (16), Hydroxyzine (5), Cyproheptadine (4), Dimenhydrinate (3), Diphenhydramine (2), Brompheniramine (1)
NSAIDs	9 (3%)	Naproxen (3), Piroxicam (3), Ibuprofen (2), Meloxicam (1)
TCAs	6 (2%)	Amitriptyline (4), Nortriptyline (2)

NSAIDs, Non-Steroidal Anti-Inflammatory Drugs; TCAs, Tricyclic Antidepressants

A segmented analysis of PIM prescriptions across various hospital phases (Table 3) showed 90 instances before admission, involving 85 patients, 34 during hospitalisation, and 11 upon discharge. In the pre-admission phase, BZDs led with 44 prescriptions, succeeded by first-generation antihistamines, NSAIDs, and TCAs. During the inpatient stage and for take-home medications, first-generation antihistamines were the most prescribed PIM class, closely followed by BZDs.

**Table 3.** Patterns of PIM medications in different hospital phases.

PIM Category	Before Admission	During Admission	Home Medication
Benzodiazepines	44	14	4
First-Generation Antihistamines	31	15	5
NSAIDs	9	2	1
TCAs	6	3	2
**Total**	**90**	**34**	**11**

NSAIDs, Non-Steroidal Anti-Inflammatory Drugs; TCAs, Tricyclic Antidepressants.

### Factors associated with PIM use in hospitalised elderly patients

Table 4 provides a detailed comparison of various variables among elderly patients with and without PIM prescriptions. Notably, age and the number of medications taken before hospital admission were significantly different between the two groups. Patients with PIM prescriptions were, on average, younger than those without (75.89 ± 8.60 vs 78.60 ± 8.15 years; p=0.048). Additionally, they took more medications before hospital admission compared to those without PIM prescriptions (12.2 ± 6.57 vs 11.0 ± 7.10; p=0.002). Other variables, such as body mass index, length of hospital stay, the number of comorbidity groups, the number of medications during hospital admission, and the number of home medications, showed no significant differences between the groups (see Table 4 for statistical details).

**Table 4.** Comparison of variables between patients with and without PIM prescriptions.

Variable	With PIM prescription	Without PIM prescription	p-value*
Age (years)	75.89 ± 8.60	78.60 ± 8.15	0.048
BMI (kg/m²)	22.2 ± 5.56	21.4 ± 4.81	0.905
Hospital Stay (days)	15.8 ± 23.08	15.7 ± 18.40	0.258
No. of Comorbidity	5.4 ± 2.59	5.8 ± 2.73	0.308
No. of Pre-Admission Meds	12.2 ± 6.57	11.0 ± 7.10	0.002
No. of During Admission Meds	17.8 ± 10.56	15.4 ± 7.81	0.277
No. of Home Meds	8.5 ± 4.41	8.0 ± 4.31	0.258

*p < 0.05 for statistical significance. Data: Mean ± SD. PIM: Potentially Inappropriate Medication, BMI: Body Mass Index (kg/m²), Pre-Admission Meds: Number of medications before admission, Admission Meds: Number of medications during admission, Home Meds: Number of prescribed home medications.

Table 5 details the outcomes of a multiple binary logistic regression analysis, examining the relationship between various independent variables and PIM prescription in elderly patients. After adjusting for confounding variables such as healthcare schemes, the results indicate that age and the number of comorbidities were significantly associated with PIM prescription. Specifically, for every one-year increase in age, the odds of receiving a PIM prescription increased by 4.8% (adjusted OR 1.05, 95% CI 1.01–1.09, p=0.01). For every one-unit increase in the number of comorbidities, the odds of receiving a PIM prescription increased by 16.2% (adjusted OR 1.16, 95% CI 1.02–1.32, p=0.021). Additionally, patients taking at least 5 medications are more likely to receive PIM prescriptions compared to those taking fewer medications (adjusted OR 3.77, 95% CI 1.15–12.35). A similar trend was observed in patients taking at least 15 medications compared to those taking fewer medications (adjusted OR 2.84, 95% CI 1.48–5.46). No significant associations were identified for other variables such as sex, body mass index, healthcare scheme, or use of herbs and supplements.

**Table 5.** Factors associated with PIM prescription in hospitalised elderly patients.

Variable	Adjusted OR	95% CI	p-value*
Age	1.048	1.008 - 1.089	0.01
No. of comorbidities	1.162	1.023 - 1.321	0.021
No. of medications before admission	0.265	0.081 - 0.866	0.028
No. of medications during admission	0.352	0.183 - 0.677	0.002
No. of home medications	0.844	0.418 - 1.706	0.637
Use of herb and supplement	1.342	0.625 - 2.883	0.450

* p < 0.05 for statistical significance.

Further analysis revealed a significant association between PIM use during admission and length of hospital stay (OR 3.32, 95% CI 1.50–7.33, p=0.003). However, PIM use did not significantly affect the likelihood of hospital readmission (OR 0.93, 95% CI 0.51–1.67, p=0.92).

## Discussion

Our study enriches the existing literature on PIM prescriptions in elderly patients by exploring their associations with clinical outcomes, as delineated by the 2019 Beers criteria. Consistent with prior research (Gallagher et al., 2011; O'Mahony et al., 2015), we found a high prevalence of PIM prescriptions among the elderly, particularly benzodiazepines (Davies & O'Mahony, 2015) and first-generation antihistamines (Cho et al., 2018). Additionally, our findings indicate that age and the number of comorbidities were significantly associated with PIM prescription, corroborating earlier study. (Kim et al., 2017)

Although the impact of PIMs on hospital length of stay and readmissions remains inconclusive according to systematic reviews and meta-analyses (Hyttinen et al., 2016; Mekonnen et al., 2021), our study offers additional evidence. Specifically, we observed a significant association between PIM use during hospitalisation and extended length of stay, while no substantial impact on readmission rates was observed.

A notable finding in our study is a marked reduction in the prevalence of PIMs from the time of admission to discharge, suggesting that hospitalisation may offer an opportunity to optimise medication regimens in the elderly. The primary source of PIMs was the continuation of pre-admission medications, supplemented by additional prescriptions during the hospital stay. We also documented rare cases in which PIMs from the same class were initiated during hospitalisation. Although our study design did not capture the specific reasons for these concurrent prescriptions, this highlights the need for further research and the potential for system-level interventions. Such measures could enhance medication safety by facilitating the early detection of PIMs and ensuring consistent medication management during a patient's hospital stay. Our findings advocate for a systematic approach to support elderly patients experiencing polypharmacy, defined as taking five or more medications, with complex health conditions.

Nevertheless, our study is not without limitations that warrant cautious interpretation of the findings. Firstly, the design of our study, with its emphasis on PIM prevalence and predictive factors of PIM use, may have limited the depth of analysis regarding the impact of PIMs on readmission rates. Although our findings did not demonstrate a significant effect, this should not detract from the potential clinical relevance of PIMs in patient outcomes. Recognising the limitations posed by our modest sample size, we recommend further research with expanded cohorts to gain a better understanding of the implications of PIM use on readmissions. Secondly, the study did not account for all conceivable confounding variables, such as healthcare provider influences, which could have a bearing on PIM prescription trends. Lastly, the study's scope was confined to four specific classes of PIMs, thus limiting the broader applicability of our conclusions.

## Conclusion

In summary, our study shows that patients on five or more pre-admission medications are likelier to receive PIMs, particularly benzodiazepines and first-generation antihistamines. Age and multiple comorbidities elevate this risk. PIM use extends hospital stays but doesn't affect readmission rates. Furthermore, our findings highlight the persistent challenge of PIMs from admission through discharge. Addressing this challenge requires a streamlined hospital policy for medication therapy management, empowering pharmacists to flag PIMs and collaborate with physicians to tailor treatment plans for geriatric patients with complex health profiles.

**List of abbreviations**
BZDs:  benzodiazepines
NSAIDs:  non-steroidal anti-inflammatory drugs
PIMs:  potentially inappropriate medications
TCAs:  tricyclic antidepressants

## Declarations

### Ethics approval and consent to participate

The study was approved by the Institutional Review Board, Faculty of Medicine, Chulalongkorn University on 26th September 2019 (COA No. 1503/2019 and IRB No. 561/62).

## Supplementary Material

Supplemental Material

## Authors’ information

All the authors of this study are full-time faculty members in the Department of Pharmacy Practice at the Faculty of Pharmaceutical Sciences, Chulalongkorn University, Bangkok, Thailand. Additionally, we serve as ward pharmacists at King Chulalongkorn Memorial Hospital, actively participating in pharmaceutical care services.
