# Supplementary material for: Prevalence and determinants of potentially inappropriate medications in elderly inpatients in Thailand: a retrospective observational study based on the 2019 Beers criteria
Source: J Pharm Policy Pract. 2023 Dec 14;17(1):2285958. doi: 10.1080/20523211.2023.2285958 (PMC10775711; doi:10.1080/20523211.2023.2285958)
Supplement: Supplemental Material [file JPPP_A_2285958_SM4823.docx]

**Additional file 1**: Table S1. Patient characteristics - additional variables

| **Characteristic** | **Number (%)** |
| --- | --- |
| Sex |  |
| Male | 150 (50) |
| Female | 150 (50) |
| Body mass index (kg/m²) |  |
| <18.50 | 66 (22.00) |
| 18.50-22.9 | 111 (37.00) |
| ≥23.00 | 84 (28.00) |
| No data | 39 (13.00) |
| Marital status |  |
| Single | 32 (10.67) |
| Married | 225 (75.00) |
| Divorced | 5 (1.67) |
| No data | 38 (12.67) |
| Occupation |  |
| Government officer | 42 (14.00) |
| Public enterprise officer | 5 (1.67) |
| Worker | 28 (9.33) |
| Housewife | 55 (18.33) |
| Business owner | 26 (8.67) |
| Other | 144 (48.00) |
| Health care scheme |  |
| Civil Servant Medical Benefit Scheme | 123 (41.00) |
| Universal Coverage Scheme | 104 (34.67) |
| Social Security Scheme | 0 (0.00) |
| Other | 73 (24.33) |
